# Abstracts from Foreign Journals

**Published:** 1902-03

**Authors:** 


### ABSTRACTS FROM FOREIGN JOURNALS.

### FRENCH.

UNDER THE DIRECTION OF S. J. J. HARGER, V.M.D.,
PHILADELPHIA, PA.

PYRAMIDON. Pyramidon is a derivative from antipyrine. It is a yellowish-white powder, insipid, soluble in water, and has properties analogous to antipyrine, but is three times more active; its effects are slower, but of longer duration. Besides preserving the antipyretic virtues, it has the same influence upon the nervous system; but this action is not accompanied by any secondary symptoms, such as malaise and vomiting. In man it is given in doses of 0.25 to 0.60 grammes daily, in solution or in tablets.—*Rec. de Med. Vet.*

CLITORIDECTOMY FOR INCONTINENCE OF URINE. Dr. Gesson reports the following case: Mare, twenty-eight years old, very nervous, with irresistible desire to urinate about every half-hour, the bladder emptying itself immediately. Remembering that the bladder and the clitoris receive their nerve-supply from the same source, and the clitoris being very sensitive and always in a state of erection, I decided upon excision of this organ, as in nymphomania. The trouble disappeared immediately.—*Archiv. Med. d'Hugers.*

ADENOMA OF THE MAMMARY GLANDS. Adenoma, very frequent in the dog and cat, is divided into *true adenoma*, *fibro-adenoma*, and *myxo-adenoma*. In man, Delbet considers these tumors the result of chronic inflammation; they are of slow evolution, non-metastatic, and without tendency to recedivity.

*True Adenoma.* These are formed of epithelial masses supported by a delicate connective-tissue stroma. They are extremely rare. Leblanc has seen only one authentic case.

*Fibro-adenoma.* These are frequent and may affect one or several glands; they may become larger than a fist. By reason of their weight they become pediculated, contused, and ulcerate and suppurate. They are of firm consistency; small cystic formations give them a mammellated appearance; their surface, on section, is grayish-white, fasciculated, and shows cystic cavities containing a dark, viscid liquid. Histologically, the adeno-fibromas are formed of trabeculæ of fibrous tissue, which surround isolated or agglomerated nests of epithelial cells.

*Vegetating fibro-adenomas* are the same as the preceding, in which more or less voluminous vegetations develop on the surface of the cystic walls.

*Myxo-adenoma.* These are of soft and encephaloid consistency, and consist of mucoid tissue in which are distributed a few glandular neoformations. Cysts have never been seen. They are more rare than the fibro-adenoma, and are always encapsulated.—*Rec. de Med. Vet.*

Two Cases of Arytenectomy. Detonte reports two successful operations. The surgical procedure was that usually described. The disease existed for several years in both instances, and the horses were useless for work. Two months after the operation in the first case, and three months in the second, all evidence of roaring under severe exercise had disappeared. The one was a half-bred and the other a Belgian draught-horse.—*Rec. de Med. Vét.*

Mumps in the Dog. In the dog this specific inflammation of the salivary glands, especially the parotid, mentioned by Schüssele and Hertwig, is very rare. Two instances mentioned by Whittaker and Busquet indicate that this disease in the dog may be of human origin. The following conclusions have been drawn: 1. The dog is susceptible to mumps. 2. The disease is transmissible from one dog to another. 3. In the diseased animal there is found a micrococcus which grows in the saliva in the form of a diplostreptococcus analogous to or identical with that found in mumps in man by Ferré and Busquet in 1895, and in the blood in the form of a diplococcus analogous or identical with that described by Laveran and Cotini in the same disease in man in 1893.—*La Presse Med.*

Treatment of "Cold" Shoulder Lameness. After a clinical experience with the individual internal remedies and external applications ordinarily used in shoulder lameness *à frigore* (cold), and especially muscular rheumatism, Trinchera, of the Veterinary School of Milan, recommends the following combination: Sod. salicyl., 20 grammes, or quin. salicyl., 4 grammes; pot. iodid., 5 grammes; tr. aconit., 3 grammes, or ext. aconit., 25 centigrammes; inf. aromat., 800 grammes. This preparation is given once daily in one dose.

The local application consists of liquor ammonia, 500 grammes; essence of turpentine, 100 grammes, and camphorated alcohol, 200 grammes. This is applied to the shoulder until the skin becomes scabby.

I have also experimented with subcutaneous injections of a saturated salt solution, 10 to 20 grammes, into the shoulder. This is most efficacious when combined with the above antirheumatismal mixture.—*La Clinica Veterinaria.*

Intravenous Injections of Corrosive Sublimate in Foot-and-Mouth Disease. This is the heading of an interesting article by Degire, who reviews the treatment of Dr. Baccelli, Minister of Agriculture of Italy. The latter borrowed this method from his clinical experience in man. It consists in the intravenous injections of a solution of bichloride of mercury.

Foot-and-mouth disease was very prevalent in Italy. Municipal veterinarian Croce treated fifty-two cases, and all rapidly recovered; two, but rarely three, injections were necessary. Twenty-six cases were treated in Sardinia, where the disease was fatal, with the same success. In many of the other provinces where the disease was wide-spread the result was the same. According to the Italian reports, the results were "nothing less than marvellous." The observers of Bacelli's treatment have concluded :

1. The intravenous injection of corrosive sublimate rapidly ameliorates the general state of the animal affected with aphthous fever.

2. It reduces the temperature in a very short time; in one case 8.5 in five hours.

3. It prevents, or in large measure reduces, the loss of milk.

4. The local symptoms become abortive, remain limited, and assume a favorable character in a very short time.

This treatment is said to be efficacious in all stages of the disease, and is especially indicated during the prodromic period.

The Minister of Agriculture of Belgium prescribed the above treatment in aphthous fever existing in Belgium, and with good success.

The *solution* employed is artificial serum, the normal salt solution, 7 to 1000. With this serum a 1 per cent. solution of corrosive sublimate is made. The dose is 2 to 4 c.c. in one injection for calves, 4 to 6 c.c. for adults, and 6 to 8 c.c. for bulls and steers. These doses can be doubled without danger. In order to prevent any irritating action upon the vessels, 20 to 30 c.c. of serum may be used. One or two injections are given daily.

The *technique* of the operation is the same as in other injections of this kind. A glass syringe is to be preferred, on account of the corrosive action of the mercury upon metals. The skin should be shaved, washed first with soap and water and then with a bichloride solution.

The veins selected are the jugular and the subcutaneous abdominal (milk) vein ; the former is preferred.

The animal is secured by tying the head to a post or tree with a rope passed around the base of the horns.

Intratracheal and subcutaneous methods have also been used, but with less gratifying results.—*Ann. de Med. Vet.*

[In the last (January) number of the *Sch. Arch. f. Thierheilkunde*, Strebel reports some careful and extended experiments made by him for the Swiss Government for the purpose of testing the alleged discovery of Bacelli in regard to the prevention and cure of foot-and-mouth disease by the intravenous injection of corrosive sublimate solution. Strebel reports that the drug has no prophylactic or curative effect.]

Comparative Experiments on the Pathogenic Action for Animals, Especially those of the Bovine Species, of the Tubercle Bacilli of Bovine and Human Origin. One culture of bovine origin derived from tubercle in the lung was employed. Five cultures of human tubercle were used. One was from a tuberculous kidney, one from tuberculosis of a sheath of a tendon, one from sputum, one from pulmonary tubercle of a child, and one from pulmonary tissue. The method of injection was intravenous, except in those cases where it seemed to offer difficulties, when the intra-abdominal method was used. Animals experimented on were goats, sheep, cattle, one horse, monkeys, and dogs. For all animals except the horse the inoculations were comparative. There being only one horse, bovine culture was used. The goats, sheep, and cattle inoculated with all cultures became tuberculous, as proven by the macroscopical lesions. The inoculations of test animals such as guinea-pigs, and the presence of tubercle bacilli, were shown by microscopical examinations of the organs and by the existence of the histological alterations of tuberculosis as well as the bacilli themselves in sections. The animals were all tested with tuberculin before the experiment, and in many cases the existence of tuberculosis was proven by the same test following the inoculation.

We are able to state that seven cattle—namely, two calves of six months, three steers of two years, and one of one year and a half, and one calf of seven or eight months—all became tuberculous by the injection of human tubercle bacilli; consequently all cattle subjected to the experiment were infected. The disease was grave and wide-spread only in one animal, in four others it showed regressive tendencies, and in the other two it was progressive. In the two cases inoculated with bovine bacilli a rapid infection took place, localized mainly in the lung, which led rapidly to death. Thus anatomy, histology, and bacteriology agree in showing that the two kinds of bacilli, in spite of their different origin, can cause tuberculosis in cattle, but from bovine bacilli the disease is grave in character and acute in progress, while that due to the human bacilli is usually more benign and chronic; in other words, the bovine bacillus is more virulent for cattle than is the human bacillus. This same difference in virulence was shown for goats as well as sheep, and also for dogs and monkeys. Our experiments show that we can produce tuberculosis in cattle, sheep, goats, dogs, and monkeys with bacilli of human origin; but with the bacilli of bovine origin we produce in the same animal a more grave disease.

*Conclusions.* I consider myself justified in drawing the following conclusions:

1. The human tubercle bacillus is capable of producing tuberculosis in cattle.

2. The human tubercle bacillus can also produce tuberculosis in other domestic animals—sheep, goats, dogs, and monkeys.

3. The tuberculosis produced in these animals by the human bacillus is generally less grave than that caused by bovine bacillus.

4. We must admit, consequently, that the bovine bacillus possesses a higher virulence than the human.

5. We cannot but believe that this superior virulence of the bovine bacilli—a superiority which is manifest in comparative experiments on cattle, sheep, goats, dogs, and monkeys—will show itself also for man.

6. It follows that man as a factor in the infection of cattle with tuberculosis has much less importance than cattle considered as a factor of infection for man.

7. Consequently bovine tuberculosis from the point of view of human health merits more attention than has been accorded to it heretofore.—Abstract of article by Dr. D. A. de Jong, of Leyden, in *La Semaine Medicale*, January 15, 1902.

GERMAN.

In the *Zeitschrift für Fleisch und Milch Hygiene* for February, 1902, Dr. Kurt Mueller, surgeon, of Effort, makes a contribution to the tuberculosis question. He described two cases of tuberculosis that came under his care. One ten years ago, and the other four years ago.

In both instances the subjects were young healthy men of clean family history, working as butchers. In each case a tendon of the hand was cut across, opening a synovial sheath, the wounds being received, according to the reports of the patients, while at work on tuberculous cattle. An operation was performed in each case, and it was found that the synovial sheath was full of slimy fluid, the wall of the sheath was thickened, as was the tendon itself, and upon them were great numbers of yellow nodules, which were shown by microscopical examination to be tubercles. These tubercles were placed very thickly in the immediate vicinity of the scar, but gradually became fewer at a distance from the scar. In one case the trouble extended from the finger to the forearm, and even at the muscular attachment of the tendon there was still some evidence of tuberculosis. In the second case the tendon was attached to the sheath, and the whole process was confined to a distance of 10 cm. Microscopical examination of the excised and curetted tissue showed the presence of tubercle bacilli.

Both of these men were, so far as could be determined, entirely free from tuberculosis in other parts of the body. Following the operation the wounds healed and the men remained healthy during the time they continued under observation. In considering the history of these cases and the conditions found, Dr. Mueller has no doubt that his patients were inoculated with tuberculosis from cattle.

The budget of the State of Prussia for 1902 contains some figures of interest to veterinarians in this country. Two of the six German veterinary schools (Berlin and Hanover) are in Prussia. For the support of these schools and for the control of the contagious diseases of animals 1,353,974 marks (about $338,400) are appropriated. For the encouragement of cattle breeding, 949,420 marks (about $237,355) are set aside. These appropriations can scarcely be compared to the appropriations to the United States Bureau of Animal Industry, because they are for purposes almost wholly different, and are not made from the Imperial Treasury, but from the funds of the State (Kingdom) of

Prussia. None of the expense of meat inspection, for example, which forms such a large part of the cost of the Bureau of Animal Industry, falls upon the above-named funds, for meat inspection is paid for by the smaller political divisions, the cities and towns.

When these appropriations are compared to those of any single American State or any group of States, the great value placed upon veterinary education and services in Germany, as compared to this country, is at once apparent.

